# Impact of delayed cord clamping and minimally invasive surfactant administration on outcomes in premature infants with neonatal respiratory distress syndrome at less than 30 weeks gestation: a NICU quality improvement study

**DOI:** 10.3389/fped.2025.1686362

**Published:** 2025-11-18

**Authors:** Yijia Zhang, Hui Zhang, Yahui Zhang, Xinyue Li, Zheng Yan, Jinghui Zhang, Hua Zhang, Meihua Piao, Tongyan Han

**Affiliations:** 1 Department of Pediatrics, Peking University Third Hospital, Beijing, China; 2 Research Center of Clinical Epidemiology, Peking University Third Hospital, Beijing, China

**Keywords:** delayed cord clamping, minimally invasive surfactant administration, respiratory distress syndrome, preterm infants, outcomes

## Abstract

**Background:**

Preventing early invasive mechanical ventilation (IMV) in preterm infants is critical for reducing bronchopulmonary dysplasia (BPD) and improving outcomes. While delayed cord clamping (DCC) enhances cardiopulmonary stability and minimally invasive surfactant administration (MISA) reduces IMV dependence, evidence on their combined efficacy in extremely preterm infants (<30 weeks gestation) with neonatal respiratory distress syndrome (NRDS) remains limited. We hypothesize that integrating DCC with MISA will synergistically reduce BPD incidence compared to immediate cord clamping (ICC) with either MISA or tracheal intubation-based surfactant delivery.

**Methods and design:**

This is a single-center quality improvement study evaluating three treatment regimens for neonates with NRDS born at a gestational age of <30 weeks: (1) Retrospective data collection of cases treated with the conventional approach of ICC + tracheal intubation between 2017 and 2020 (*n* = 222); (2) Retrospective data collection of cases treated with ICC + MISA between 2021 and 2025 (*n* = 222); (3) Planned collection of medical records of cases treated with DCC + MISA between 2025 and 2027 (*n* = 74).The study aims to assess the incidence of BPD and survival outcomes associated with each regimen. Meanwhile, it will compare the short-term efficacy, safety, and long-term outcomes of these three treatment strategies, thereby providing valuable evidence for clinical treatment decision-making.

**Discussion:**

Current research indicates that both DCC and MISA positively impact the prognosis of very premature infants and help reduce the incidence of BPD. However, there is limited research on whether the combined use of DCC and MISA can further improve survival rates and reduce the incidence of BPD in this vulnerable population. Our NICU has gradually implemented MISA in respiratory management since 2021 and adopted cord management with DCC since 2025. This study retrospectively analyzes data from previous groups: those receiving ICC with MISA, and those receiving ICC with tracheal intubation. We will compare this with prospective data from the DCC combined with MISA group to assess differences in BPD occurrence, other complications and overall outcomes. This study will also collect and analyze the annual application rates and operational success rates of MISA and DCC, so as to promote quality improvement in the NICU. Through this study, we aim to determine whether the combination of DCC and MISA offers greater benefits in improving the prognosis of very premature infants, ultimately providing a stronger foundation for early respiratory and circulatory management strategies for infants born before 30 weeks.

**Trial Registration:**

https://register.clinicaltrials.gov, Identifier:NCT07092319.

## Background

With the rapid advancement of neonatal intensive care technology, the survival rate of premature infants born at less than 30 weeks gestation continues to rise. However, the immaturity of various organ systems leads to a high incidence of complications, and long-term outcomes can often be less than optimistic (1). Consequently, enhancing both short-term and long-term prognoses for these infants through quality improvement initiatives has become a prominent focus in recent clinical research.

BPD is a critical complication in very premature infants, significantly affecting their future survival, as well as their respiratory and neurological development. Preventing and reducing the incidence of BPD is crucial for improving the overall prognosis of these infants (2). Ensuring postpartum respiratory and circulatory stability while minimizing exposure to early invasive mechanical ventilation is essential for preventing and reducing the incidence of BPD.

The management of respiratory and circulatory conditions in preterm infants begins with DCC in the delivery room. Compared to ICC, DCC facilitates the transfer of blood from the placenta to the infant, enhancing the adaptability of vital organs such as the heart, brain, and lungs. This practice supports a smoother transition from intrauterine to extrauterine life, promotes the conversion of respiratory circulation, and can reduce preterm infant mortality by 30%. Additionally, DCC decreases the incidence of complications such as intraventricular hemorrhage, necrotizing enterocolitis, and late-onset sepsis. It also increases blood volume, minimizes the need for blood transfusions, enhances iron storage, and lowers the risk of long-term neurodevelopmental issues. Therefore, DCC emerges as a viable strategy to enhance the perinatal prognosis of preterm infants (3–7).

However, there are limited reports in China regarding the implementation of DCC in premature infants born at less than 30 weeks gestation. Our hospital began adopting DCC for preterm infants in 2025 and has developed and standardized the operational procedures for this practice. However, relevant clinical studies have yet to be conducted. NRDS is a condition resulting from a deficiency of pulmonary surfactant in the alveoli. It is the most common respiratory issue encountered by very premature infants, who often experience progressively worsening respiratory distress shortly after birth. Treatment for NRDS involves respiratory support and the administration of exogenous PS. Traditionally, this is done through tracheal intubation. However, recent clinical studies have demonstrated that MISA offers several advantages over tracheal intubation (8). MISA effectively reduces the need for invasive mechanical ventilation within the first 72 h and plays a significant role in lowering the incidence of BPD (9).

With advancements in DCC and MISA technology in NICU settings, these approaches have become the preferred options for very premature infants who are breathing spontaneously at birth. To further assess the safety and effectiveness of DCC combined with MISA in our hospital's NICU for premature infants born at less than 30 weeks gestation, we propose this quality improvement study. The study aims to compare the incidence and prognosis of BPD and other complications before and after the implementation of DCC and MISA in the NICU.

Our hospital comprehensively implement the MISA since 2021 and has gained extensive clinical experience in treating NRDS in premature infants using this method. We previously conducted a prospective multicenter study comparing MISA with tracheal intubation for surfactant administration shortly after birth. The results indicated that the incidence of BPD and hemodynamically significant patent ductus arteriosus (hsPDA) was lower in the MISA group, highlighting its suitability for premature infants born at less than 30 weeks gestation (10, 11). Our hospital has gradually implemented DCC for premature infants prior to this research and has established a comprehensive protocol for its application in the delivery room and during surgical procedures. As a result, our NICU is well-equipped to perform DCC combined with MISA for infants born at less than 30 weeks gestation. Based on previous research findings, this study specifically targets premature infants under 30 weeks, a group that exhibits a relatively high incidence of BPD. By monitoring the respiratory and overall health outcomes of these infants following the application of DCC combined with MISA, we aim to evaluate the effectiveness of this NICU quality improvement measure in enhancing the prognosis of very premature infants.

## Methods

### Study design

For preterm infants with a gestational age of <30 weeks diagnosed with NRDS, along with the continuous advancement of diagnosis and treatment in the NICU, they were divided into 3 phases based on time and treatment methods:

1. From 2017 to 2020, retrospective data were collected from infants treated with ICC combined with tracheal intubation for pulmonary surfactant administration;
2. In 2021, after the promotion of MISA in the NICU, retrospective data were collected from 2021 to 2025 of infants treated with ICC combined with MISA;
3. Starting from 2025, as the NICU has popularized the operational procedures for DCC in preterm infants, a prospective cohort study is planned to be conducted, and data will be collected from infants treated with DCC combined with MISA.

**Figure 1:**
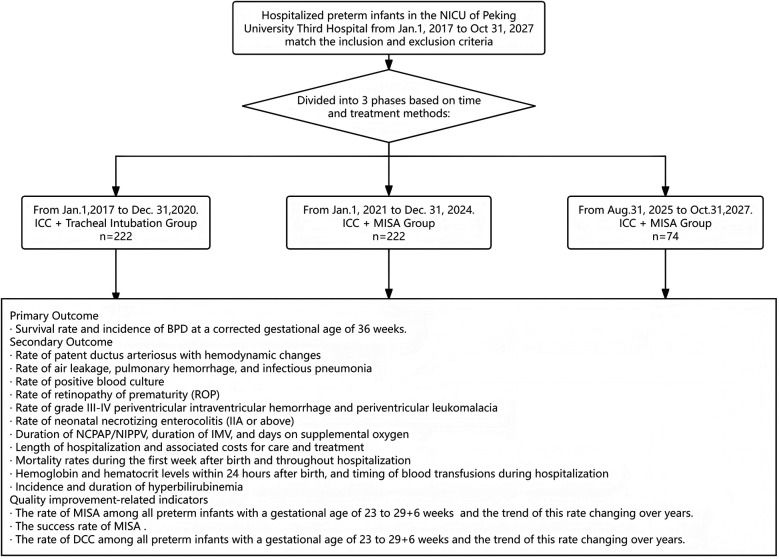
Flow chart nof the study. This study is a single-center ambispective cohort study involving hospitalized preterm infants in the NICU of Peking University Third Hospital from Jan. 1, 2017, to Oct. 31, 2027. The subjects from Jan. 1, 2017, to Dec. 31, 2024, were included in the retrospective cohort study, while a prospective cohort study was conducted from Aug. 31, 2025 to Oct. 31, 2027.

This study evaluated the incidence of BPD, survival status, and other comorbidities associated with each treatment regimen, so as to provide references for the formulation of clinical decisions regarding treatment option selection. The operational process of this study is illustrated in Figure 1.

### Participants

All infants participating in the study were selected using the same inclusion and exclusion criteria.

#### Inclusion criteria

① Infants of 23–29 + 6 weeks gestational age.
② Born in Peking University Third Hospital, having spontaneous breathing after birth;
③ Diagnosed as NRDS; NRDS is defined as progressive dyspnea that occurs after birth, with or without imaging evidence, excluding other causes of dyspnea.

#### Exclusion criteria

① Premature infants who have received tracheal intubation prior to the use of PS due to resuscitation or other reasons shortly after birth;
② Premature infants with identified or confirmed congenital abnormalities (such as fetal edema, chylothorax, or neuromuscular disease) or severe birth complications (including hemorrhagic shock, air leakage, or early-onset sepsis) that impact respiratory function;
③ Newborns who discontinue treatment or have incomplete information;
④ Newborns involved in other intervention research studies.

### Research grouping

Patients were divided into three groups based on the study's time phases and the differences in treatment they received after birth:

#### ICC+tracheal intubation group

From Jan.1, 2017 to Dec. 31, 2020. Umbilical cord clamping occurs within 20 s after birth. Infants are diagnosed with NRDS and receive a therapeutic dose of PS via tracheal intubation within the first 12 h after birth.

#### ICC+MISA group

From Jan.1, 2021 to Dec. 31, 2024.Umbilical cord clamping is performed within 20 s after birth. Infants are diagnosed with NRDS and receive a therapeutic dose of PS via MISA within the first 2 h after birth.

#### DCC+MISA group

From Aug. 31, 2025 to Oct. 31, 2027. Infants without contraindications to DCC, as determined by prenatal evaluation, receive immediate non-invasive respiratory support after birth. DCC is successfully performed for 60 s, followed by a diagnosis of NRDS. A therapeutic dose of PS is administered via MISA within the first 2 h after birth.

### Quality control

This trial adheres to Good Clinical Practice (GCP) standards and meets the requirements set by the Ethics Committees. It is conducted under the supervision of the Ethics Committees at the participating institutions. A Data Safety Monitoring Board (DSMB) will be established, comprising two independent neonatologists and an independent statistician. The DSMB will have designated review points to assess trial progress and safety.

In clinical practice, neonatologists receive training on MISA and DCC before rotating to the NICU, and undergo regular assessments. This ensures that there are neonatologists proficient in MISA and DCC on duty 24 h a day, who can perform the corresponding clinical procedures for preterm infants delivered at any time. Upon entering the NICU, nasal continuous positive airway pressure (NCPAP) or nasal intermittent positive pressure ventilation (NIPPV) will be initiated as the first line of non-invasive respiratory support. During MISA, the infant is placed in an incubator with the temperature set at 37°C. The infant's limbs are properly swaddled to soothe them, preventing crying and injury. A laryngoscope is used to expose the glottis, and a thin catheter is inserted under the maintenance of non-invasive ventilation support, with the insertion depth calculated as (infant's weight in kg) + 5.5/6 cm. Pulmonary surfactant (Curosurf, 200 mg/kg or Calf Pulmonary Surfactant for Injection, 100 mg/kg) is slowly instilled, with the total administration time being approximately 3 min. If the infant cries or experiences a drop in heart rate or oxygen saturation, the administration is paused; it is resumed after the infant's condition stabilizes until the full dose is administered.

In collaboration with the obstetrics department, operational protocols for DCC during deliveries in the labor room and operating room have been developed. Monthly training on neonatal resuscitation for asphyxia and DCC is provided to midwives and obstetricians, ensuring that the condition of newborns is quickly evaluated during delivery and that safe DCC is performed for those without contraindications.

### Outcome measures

#### Primary outcome measure

The primary outcome is the survival rate and incidence of BPD at a corrected gestational age of 36 weeks.

Diagnosis and classification of bronchopulmonary dysplasia will follow the International Workshop BPD classification standards proposed by the US NICHD in 2019 (12).

#### Secondary outcome measures

The secondary outcomes are as follows:

1. Rate of hsPDA
2. Rate of air leakage, pulmonary hemorrhage, and infectious pneumonia
3. Rate of late-onset sepsis with positive blood culture
4. Rate of retinopathy of prematurity (ROP)
5. Rate of grade III-IV periventricular intraventricular hemorrhage (IVH) and periventricular leukomalacia (PVL)
6. Rate of neonatal necrotizing enterocolitis (IIA or above)
7. Duration of NCPAP/NIPPV, duration of IMV, and days on supplemental oxygen
8. Length of hospitalization and associated costs for care and treatment
9. Mortality rates during the first week after birth and throughout hospitalization
10. Hemoglobin and hematocrit levels within 24 h after birth, and timing of blood transfusions during hospitalization
11. Incidence and duration of hyperbilirubinemia

#### Quality improvement-related indicators

1. The rate of first-time pulmonary surfactant administration via MISA among all preterm infants with a gestational age of 23–29 + 6 weeks admitted to the NICU and the trend of this rate changing over years.
2. The success rate of MISA (defined as the completion of pulmonary surfactant administration under non-invasive support, without early termination of administration or switching to tracheal intubation for administration).
3. The rate of DCC among all preterm infants with a gestational age of 23–29 + 6 weeks admitted to the NICU and the trend of this rate changing over years.

### Data collection and diagnoses

Demographic data will include gestational age, birth weight, sex, multiple births, antenatal corticosteroid use, mode of delivery, maternal complications, Apgar score, and cord blood gas levels. Clinical data will encompass survival rates, required doses and timing of surfactant administration, blood gas measurements on the first day after birth, need for mechanical ventilation during hospitalization, duration of NCPAP/NIPPV, days on supplemental oxygen, hospitalization costs, and morbidity of BPD, pneumothorax, nasal trauma, pulmonary hemorrhage, hsPDA, IVH, PVL, blood culture-confirmed late onset sepsis, NEC, and ROP.

Pneumothorax will be diagnosed by the presence of air in the pleural cavity in a chest x-ray. Pulmonary hemorrhage will be diagnosed based on the gushing of bloody fluid from the upper airway or endotracheal intubation and when the chest x-ray is consistent with the relevant clinical findings. The diagnosis for hsPDA will be based on clinical signs and echocardiogram. IVH and white matter injury will be diagnosed by cranial ultrasound examination, and IVH will be graded by the Papile classification system. Blood culture-confirmed sepsis will be defined as clinical sepsis with evidence of pathogens in blood culture. NEC will be graded according to the modified Bell's classification system. The diagnosis and staging of ROP will be based on retinal examination by a consultant ophthalmologist.

Pneumothorax will be diagnosed radiographically by detecting air within the pleural cavity on chest radiography. Pulmonary hemorrhage is defined as acute onset of frank blood emanating from the upper airway or endotracheal tube, accompanied by radiographic evidence concordant with clinical manifestations. The diagnosis of hsPDA will be established through integration of clinical indicators and confirmatory echocardiography (13). IVH and PVL will be identified via standardized cranial ultrasonography, with IVH severity classified according to the Papile grading system (14). Culture-confirmed late-onset sepsis requires both compatible clinical signs and isolation of pathogenic microorganisms from blood cultures. NEC will be staged using the modified Bell's classification criteria (15). ROP diagnosis and staging will be determined through comprehensive retinal evaluations performed by a certified ophthalmologist.

### Safety procedures

This study is observational in nature. Should any adverse events occur, they will be accurately documented, and appropriate medical treatment will be provided.

### Sample size calculation

Our hospital has previously conducted a prospective multicenter clinical study comparing MISA and tracheal intubation in the early stages. The results indicated that the incidence of BPD in premature infants with a gestational age of less than 30 weeks was 29% in the MISA group (*n* = 31) and 70% in the tracheal intubation group (*n* = 20). Assuming the BPD incidence rate (p_1_) in the ICC + MISA group for this study is 29%, and the incidence in the DCC + MISA group (p_2_) decreases by 15%–14%, we will set the Type I error (α) at 0.05 and the Type II error (β) at 0.2. With a sample size ratio (k) of 3:1 between the DCC + MISA and ICC + MISA groups, the sample size calculation formula is:

$$n_{2}=\frac{{\left(z_{1-a/2}+z_{1-\beta }\right)}^{2}[\;p_{1}(1-p_{1})/k+p_{2}(1-p_{2})]}{{\left(\;p_{1}-p_{2}\right)}^{2}}$$

*n*_1_ = *k* × *n*_2_.

Where z_(1−*α*/2)_ = 1.96 and _z(1−*β*)_ = 0.84.

This results in a sample size of 66 for the DCC + MISA group and 198 for the ICC + MISA group. Assuming a 10% loss to follow-up, the adjusted sample sizes will be 74 for the DCC + MISA group and 222 for the ICC + MISA group. Additionally, the sample size for the ICC + tracheal intubation group is also set at 222 cases.

### Statistical analysis

Statistical analyses were conducted using SPSS 20.0 software. Normally distributed data are presented as mean ± standard deviation, with intergroup comparisons performed using one-way analysis of variance (ANOVA) and *post hoc* tests conducted via the Student-Newman-Keuls (SNK) method for Type I error correction. Non-normally distributed data are expressed as median (Q1, Q3), and intergroup comparisons are made using the Kruskal–Wallis test.

For categorical data, counts and percentages are used for intergroup comparisons, employing the *χ*² test with continuity correction or Fisher's exact test as appropriate. The analysis of factors influencing binary outcomes will be performed using binary logistic regression, accounting for potential confounding variables. A *p*-value of <0.05 will be considered statistically significant.

To assess the robustness of the main conclusions of the study, we will conduct the following sensitivity analyses: To control for potential confounding factors as comprehensively as possible, we will include all baseline variables with intergroup differences reaching a significance level of *P* < 0.1 in the univariate analysis, together with the study grouping variable, into the multivariable Logistic regression model. This is to calculate the adjusted odds ratio (aOR) of the effect of DCC + MISA on BPD and its 95% confidence interval (95% CI).

## Discussion

With the rapid advancement of neonatal intensive care technology, NICU are facing increasing challenges. Improving the prognosis of small gestational age premature infants, aiming to achieve higher survival rates, reducing complications, and enhancing future quality of life-has become a central focus in the treatment of these infants. To address these challenges, NICU must adopt new concepts, adjust treatment policies, and implement innovative clinical procedures. DCC and MISA are prime examples of such advancements.

DCC has been utilized for term infants for many years, demonstrating positive effects on hematocrit levels and iron reserves. It is now a standard recommendation for term infants following delivery; however, its benefits for premature infants, particularly those who are extremely premature, remain unclear. Recently, an increasing number of studies have investigated DCC in premature infants, with multiple systematic reviews and meta-analyses highlighting the potential for improved outcomes through various umbilical cord management strategies (7, 16). Despite these findings, a unified global recommendation has yet to be established. A meta-analysis published in The Lancet in 2023 reaffirmed that DCC positively impacts reducing in-hospital mortality in premature infants (17). Our NICU, which has focused on treating premature infants gently for many years, began gradually implementing DCC for those without contraindications in 2023. We have developed operating procedures for the delivery and operating rooms and provided training for obstetricians and neonatologists to ensure the stable and safe application of DCC. However, due to the recent implementation, no relevant research has been conducted yet.

MISA, which involves the insertion of a thin tube into the airway to deliver pulmonary surfactant alongside non-invasive respiratory support, reduces the need for mechanical ventilation and decreases the incidence of BPD. This approach is already a common recommendation in clinical guidelines (18). As one of the early adopters of MISA in China, our hospital has previously documented its effectiveness in improving BPD outcomes and has accumulated extensive experience with MISA procedures.

DCC not only enhances the transfer of blood from the placenta to the fetus, improving hemoglobin reserves in premature infants, but it also stabilizes the establishment of pulmonary circulation in the early postnatal period. Since placental circulation provides 30%–50% of cardiac blood flow, immediate clamping of the umbilical cord can lead to a significant drop in venous return and cardiac output. DCC facilitates a smoother transition until premature infants can establish effective pulmonary ventilation and circulation. Thus, DCC serves as a crucial component of early respiratory and circulatory management for these infants. This study aims to determine whether the simultaneous application of DCC and MISA further enhances survival and prognosis in premature infants. Given our recent implementation of DCC for infants born before 30 weeks, we designed a NICU quality improvement study to evaluate the efficacy of DCC combined with MISA by comparing prospective and retrospective cohorts.

Despite the potential of this study, there are some limitations to consider. As a quality improvement study, data for the ICC and MISA and ICC and tracheal intubation groups were collected retrospectively, which may introduce biases due to variations in hospitalization years and treatment approaches. Furthermore, the retrospectively collected data are based on the diagnosis, treatment, and evaluation conducted by clinicians, and there may be variations in this process. To minimize these discrepancies, we limited the study to preterm infants from the past six years for the retrospective cohort. Additionally, both the prospective and retrospective cohorts adhered to the same inclusion and exclusion criteria to reduce intergroup differences and enhance the validity of the conclusions.

## Conclusion

If the combination of DCC and MISA proves effective in reducing BPD in premature infants born before 30 weeks and enhances their prognosis compared to the ICC and MISA or ICC and tracheal intubation groups, this finding will provide a theoretical foundation for improving the quality of care in the NICU and optimizing early respiratory and circulatory management for small gestational age premature infants.
